# The feasibility and results of a population-based approach to evaluating prostate-specific antigen screening for prostate cancer in men with a raised familial risk

**DOI:** 10.1038/sj.bjc.6602925

**Published:** 2006-01-24

**Authors:** J Melia, D Dearnaley, S Moss, L Johns, P Coulson, C Moynihan, J Sweetman, M C Parkinson, R Eeles, M Watson, and collaborating consultants

**Affiliations:** 1 Cancer Screening Evaluation Unit, Institute of Cancer Research, Brookes Lawley Building, 15 Cotswold Road, Sutton, Surrey SM2 5NG, UK; 2 Academic Unit of Radiotherapy & Oncology, Institute of Cancer Research and Royal Marsden NHS Foundation Trust, Downs Road, Sutton, Surrey SM2 5PT, UK; 3 Psychology Research Group, Institute of Cancer Research and Royal Marsden NHS Foundation Trust, Downs Road, Sutton, Surrey SM2 5PT, UK; 4 University College Hospitals and Institute of Urology, UCL, London SW1 V3JN, UK; 5 Translational Cancer Genetics Team, Institute of Cancer Research & Cancer Genetics Unit, Royal Marsden NHS Foundation Trust, Downs Road, Sutton, Surrey SM2 5PT, UK; 6 Psychological Medicine, Royal Marsden NHS Foundation Trust, Downs Road, Sutton, Surrey SM2 5PT, UK

**Keywords:** prostate-specific antigen, mass screening, family health, prostatic neoplasm

## Abstract

The feasibility of a population-based evaluation of screening for prostate cancer in men with a raised familial risk was investigated by studying reasons for non-participation and uptake rates according to postal recruitment and clinic contact. The levels of prostate-specific antigen (PSA) and the positive predictive values (PPV) for cancer in men referred with a raised PSA and in those biopsied were analysed. First-degree male relatives (FDRs) were identified through index cases (ICs): patients living in two regions of England and diagnosed with prostate cancer at age ⩽65 years from 1998 to 2004. First-degree relatives were eligible if they were aged 45–69 years, living in the UK and had no prior diagnosis of prostate cancer. Postal recruitment was low (45 of 1687 ICs agreed to their FDR being contacted: 2.7%) but this was partly due to ICs not having eligible FDRs. A third of ICs in clinic had eligible FDRs and 49% (192 out of 389) agreed to their FDR(s) being contacted. Of 220 eligible FDRs who initially consented, 170 (77.3%) had a new PSA test taken and 32 (14.5%) provided a previous PSA result. Among the 170 PSA tests, 10% (17) were ⩾4 ng ml^−1^ and 13.5% (23) tests above the age-related cutoffs. In 21 men referred, five were diagnosed with prostate cancer (PPV 24%; 95% CI 8, 47). To study further the effects of screening, patients with a raised familial risk should be counselled in clinic about screening of relatives and data routinely recorded so that the effects of screening on high-risk groups can be studied.

The effects of screening by serum prostate-specific antigen (PSA) on prostate cancer mortality are not known. The European Randomized Study of Screening for Prostate Cancer (ERSPC) (Roobol and Schroder, 2003) and the Prostate Lung Colorectal and Ovarian cancer screening trial in the US (Prorok *et al*, 2000) offer screening by the PSA test in men aged 50–69 years (with some variation in age between participating countries). In addition, in the UK ProtecT treatment trial of early-stage prostate cancer (Donovan *et al*, 2002), general practices are randomised to join either the trial in which cancers are being detected by offering men a PSA test or a control arm not entering the trial. The earliest that results on mortality from these studies will be available is 2010 (Roobol and Schroder, 2003). Even if screening reduces mortality, there remains concern about the overdiagnosis of clinically nonsignificant cancer and about the uncertain benefits of radical treatment in all men diagnosed with prostate cancer compared with active surveillance (Hardie *et al*, 2005).

The efficiency of screening might be improved by targeting moderate- to high-risk groups. Genetic risk of prostate cancer could account for up to 10–15% of cases. The relative risk of prostate cancer increases with the number of affected first- or second-degree relatives diagnosed with prostate cancer, from about two with a single first-degree relative to 8.8 with both a first- and second-degree relative (Carter *et al*, 1993; Verhage *et al*, 2004) affected. Several studies outside the UK (Bratt *et al*, 2000; Makinen *et al*, 2002; Roumier *et al*, 2004; Verhage *et al*, 2004) have investigated screening in family members with a high risk of prostate cancer, but the definitions of high risk vary according to whether only first-degree or other relatives are included.

The study aims to assess the feasibility of a population-based approach to evaluating the effects of screening for prostate cancer in first-degree male relatives (FDRs) of patients diagnosed with prostate cancer at age ⩽65 years in the UK and to study the impact of PSA testing on referral for biopsy. Feasibility was assessed by developing different methods of recruitment and studying uptake rates and reasons for non-participation at several stages during this process. Referral rates for FDRs with raised PSA levels, and the positive predictive value (PPV) of the PSA test in FDRs referred for biopsy with a raised PSA level, were also analysed. The study was not designed to investigate the efficacy of screening. The results of a psychosocial study conducted in conjunction with the screening study (refer Sweetman *et al*, 2006) are reported separately.

## MATERIALS AND METHODS

**Figure 1:**
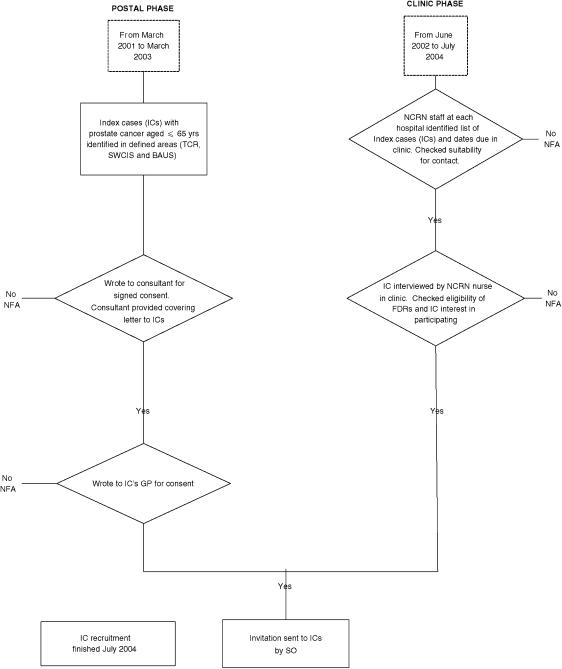
Procedures to recruit FDRs for PSA screening (IC=index case; FDR=first-degree male relative; NFA=no further action).

Procedures for consent and screening are summarised in Figure 1. The Screening Office (SO) at the Institute of Cancer Research coordinated the recruitment and data collection in both the postal phase and clinic phase.

## ETHICAL APPROVAL

The study was approved by the South London Multi-centre Research Ethical Committee (MREC January 2000).

### Study population

*Index cases* (ICs) were defined as patients living in the catchment areas of two cancer registries (London and the central and western areas of southern England) diagnosed with prostate cancer between 1 January 1998 and 31 July 2004 and aged ⩽65 years at diagnosis. All ICs had to be alive and not considered by their consultant to be seriously ill or in distress. For those ICs in the postal phase, their general practitioners (GPs) were also first approached to check on health status. *First-degree male relatives* were eligible for the screening study if they were aged 45–69 years, living in the UK and had no prior diagnosis of prostate cancer. Prior PSA measurement did not exclude FDRs from the study.

In the postal phase, ICs were identified from the Thames Cancer Registry (TCR), South West Cancer Intelligence Service (SWCIS) and the British Association of Urological Surgeons (BAUS) database. In a pilot postal phase in 2001, only FDRs of ICs identified through the TCR who lived within easy reach of London were invited to take part. Referrals in the pilot were assigned to consultant urologists in London and biopsies (only one FDR referred) were couriered to MCP for pathological diagnosis. This restriction was removed when recruitment was expanded.

The clinic phase was developed from July 2002. The study was adopted by the National Cancer Research Network (NCRN) and expanded to identify ICs from hospital records who were attending hospitals in five Cancer Research Networks: Peninsula; Avon, Somerset and Wiltshire; Dorset; South West London; and Central South Coast. Working mainly in oncology clinics, but also in some urology clinics, the NCRN nurses and urological specialist research nurses identified ICs eligible by date of and age at diagnosis. There was no standardised data source used by the staff to identify ICs; some had access to computerised patient lists giving date of birth and type of cancer. Other hospitals relied on checking patient notes at the beginning of each clinic. The years of diagnosis and time periods of data collection from each source are summarised in Table 1.

### Consent and information procedures

Signed consent to approach ICs was required from the consultants and initially from GPs (Figure 1). Delays in replies led to revisions so that GPs were notified and contacted by phone, but signed consent was not required. Signed consent was obtained from each IC and FDR. In the postal phase, MREC did not permit follow-up letters to IC non-responders or collection of data on reasons for non-participation. However, in the clinic phase, a standardised form was used to collect data on eligibility and reasons for non-participation, so that invitations were only sent to ICs with a confirmed FDR. Subsequent procedures were identical for all men. In the final year of recruitment at two hospitals (Royal Marsden Hospital (RMH) and Salisbury), a postal invitation was used to contact ICs who could not be seen in clinic before the closure date for recruitment.

In the clinic phase, the study could be discussed with the research nurse and consultant, and an information sheet about the study, which included a contact number for the SO, was given to each IC if they expressed an interest in receiving the invitation letter. In both phases, the information sheet was included with the invitation letters to both the ICs and the FDRs. First-degree male relatives were encouraged to discuss the study with their GP.

### Screening procedure

First-degree male relatives were invited to provide a blood sample for the measurement of PSA. Those who had been PSA tested/screened more than 12 months prior to contact were invited to be re-screened under the study protocol, while those who had their PSA measured within 12 months prior to the study invitation were invited to provide the result of their previous test. The SO sent each GP a venepuncture kit to collect a 7 ml serum sample for PSA testing. The sample was sent by first class post to the SO using approved packaging and was analysed at the RMH biochemistry laboratory using the Abbott AxSym analyser. The guidelines were based on the NHS Prostate Cancer Risk Management Programme recommendations (http://www.cancerscreening.nhs.uk/) and published results (Oesterling *et al*, 1993): for ages 45–59 years: refer if ⩾3 ng ml^−1^, repeat test at 6 months if 2–2.9 ng ml^−1^; for ages 60–69 years: refer if ⩾4 ng ml^−1^, repeat test at 6 months if 3–3.9 ng ml^−1^.

Each GP and FDR was notified by letter of their PSA result and whether there was a recommendation either for a re-test at 6 months or for referral. Screening started on 6th June 2001 and continued up to 31st March 2005 to reach the target sample size.

### Referral and diagnosis

When referral was recommended, the SO advised the GP about consultant urologists who were members of BAUS at the hospital routinely used by the GP. The GP made the final decision about referral after discussion with the FDR. Once referral was confirmed, the SO sent the consultant a standardised biopsy protocol and reporting procedure, based on the BAUS proforma. A minimum of eight biopsy cores (four per side), ultrasound guided, was recommended to be taken using an 18-gauge needle. Once the pathological diagnosis was received from the hospital, a pathological review was requested and conducted by MCP.

### Questionnaire data

Each FDR completed a self-administered questionnaire asking about ethnic group, using the 1991 Census categories, household composition and marital status at the time of signing consent. After providing the PSA measurement, the men also completed a questionnaire on the past history of PSA testing.

### Statistical analysis

Simple descriptive analyses were conducted using proportions and percentage distributions. The main outcome measures, uptake rates and distributions of PSA levels, were analysed by age for both ICs and FDRs. Confidence intervals for proportions were calculated using the exact method for binomial distributions. The PPV of referral was the proportion of men diagnosed with prostate cancer on biopsy out of all men with a raised PSA who were recommended by the study protocol and proceeded to be referred. The PPV of referral in the general population for PSA testing was assumed to be 20%, and that in men with familial prostate cancer to be higher at 50%. Thus, 17 FDRs with a positive PSA test were needed to have 80% power of detecting a statistically significant difference between these estimates (*P*<0.05). Assuming a positive rate of 10%, a sample size of 170 FDRs was needed.

## RESULTS

The registries and BAUS database identified a total of 1687 ICs; 1140 ICs were contacted in clinic, and 130 ICs at two hospitals, not seen in clinic, received invitation letters (Table 2).

### Measures of feasibility

#### Uptake rates by method of recruitment

In the postal phase, 48% of consultants (68 out of 143) responded, giving consent for 515 ICs to be sent invitation letters by the SO (Table 2). General practitioner's consent was obtained for 340 out of 515 ICs. An additional 12 ICs on the BAUS register were invited. Out of 352 ICs who were sent letters, 45 returned signed consent for the FDRs to be contacted. (Using this method of contact, it is not known what proportion of ICs actually had an eligible FDR.) Out of 39 FDRs who consented either to have their PSA measured or to provide a previous result and were aged 45–69 years at the time of their PSA test, 30 proceeded to provide a test result. (Of the 1687 ICs, 186 (11%) were contacted during the pilot when only FDRs living within reach of London took part, and it is not known how many ICs did not respond because of this exclusion.)

In the clinic phase, 389 of the 1140 ICs (34%) agreed in clinic to receive further information and an invitation letter, and 192 (16.8%) returned signed consent, leading to 167 (14.6%) FDRs who consented and were aged 45–69 years at the time of their PSA test (Table 2). Two FDRs were aged 44 years when they consented, and requested to have their PSA test taken when they reached 45. Of the 167 FDRs consenting, 134 proceeded to have their PSA measured and 24 provided a previous test result. Two hospitals identified a further 130 ICs who could not be seen, or had been missed, in clinic before recruitment closed. Their GPs gave permission for 117 to be contacted by letter, leading to 10 FDRs having their PSA measured and four providing a previous result (Table 2).

Most ICs provided details of one FDR (204 out of 257, 77.5%) and the remaining 53 provided details of two or more FDRs. Most FDRs were brothers, but two were sons of ICs.

#### Reasons for non-participation and characteristics of participants

Non-participation recorded in clinic phase: Of the 1140 ICs identified in clinic, seven were not approached (consultant decision), 59 refused (some adding that they had not told their family that they had cancer), 94 only had FDRs living abroad and 591 reported no suitable relative. There were 16 FDRs reported to have prostate cancer, but these data are likely to be incomplete.

Characteristics of ICs: Age at diagnosis was recorded on the database by the SO for all but two ICs identified from the cancer registries. Age at diagnosis was among the entry criteria used by the hospital staff, but some centres did not send dates of birth or diagnosis to the SO for local reasons of confidentiality (697). For 2252 ICs (76%) with known date of birth, the proportion below the age of 60 years was 41% for those identified at the outset and 45% for those agreeing to receive the invitation letter.

Characteristics of FDRs: A total of 202 FDRs provided a PSA measurement: 170 FDRs had their PSA test taken for the study and 32 provided a previous measurement. They were aged 45–69 years at the time that their blood sample was taken. Just over half of the FDRs (53%) were aged 45–59 years. All of the FDRs giving consent returned the self-administered questionnaire. Of these, 98% were white subjects, 2% were Asian or Chinese, and none were African or African Caribbean. The majority (87%) were married or co-habiting, with 6% divorced or separated, 6% single or widowed and 1% not known. Of 202 FDRs, 185 replied to the question about having had a PSA test before the one recorded in our study: 128 (69%) said that the PSA measurement provided was their first test and 57 (31%) reported having had a previous test.

#### Time to recruit

In the postal phase, the median time for ICs to reply was 10 days (range 1–441 days, 75% interquartiles 22 days). In the clinic phase, the median time for ICs to reply was 16 days (range 2–232 days, 75% interquartile 36 days). The times between the date when an IC was sent an invitation letter and the last date when a result was recorded for an FDR following the PSA test (ranging from FDR receiving a negative test result to date of biopsy) were as follows: in the postal phase, medians of 96 and 85 respectively, 75% interquartiles of 133 and 176 respectively, range 42–606 days; in the clinic phase, medians of 100 and 149 respectively, 75% interquartiles of 148 and 240 respectively, range 29–705 days.

### Prostate-specific antigen measurement

#### Sources

Most PSA measurements (164) were taken according to the study protocol and analysed at RMH. For the six PSA measurements taken for the study but analysed locally, the types of assays were recorded. Variation in measurements between these assays was checked (the National External Quality Assurance scheme, Peter White, personal communication, 2005). The six PSA levels recorded at the local laboratories were such that the decisions whether or not to refer the FDRs would have been unaffected by variation in measurement between assays. Therefore, the PSA results (170) were grouped together in the data analyses.

#### Prostate-specific antigen levels

Of the 170 PSA tests taken for this study, 17 (10%) were ⩾4 ng ml^−1^ and 23 (13.5%) were above the age-related cutoffs. Among the 23 men with raised PSA levels recommended for referral, 14% (12 out of 88) in the age range 45–59 years had levels ⩾3 ng ml^−1^ and 13% (11 out of 82) in the age range 60–69 years had levels above ⩾4 ng ml^−1^. Among the 170 men, the PSA levels ranged from 0.17 to 13.47 ng ml^−1^ in 122 men who reported no previous PSA test (this included 11 with history not known) and from 0.23 to 6.10 ng ml^−1^ in 48 men with a previous test.

Of 23 men with raised PSA, 21 agreed to be referred. The PPV of referral was 24% (95% CI 8, 47) in the 170 men whose PSA was measured by the study protocol (Table 3). The proportions referred were 11% (13 out of 122) and 17% (eight out of 48) in FDRs with no previous PSA test and in FDRs with a previous test, respectively. There appeared to be a higher PPV of referral in FDRs reporting no previous test (31%, 95% CI 9, 61) than in men with a previous test (12.5%, 95% CI 0, 53) but the numbers were small and the CI wide. Five cancers were diagnosed, all being clinically organ confined with Gleason grade distribution: one 2+2, three 3+3 and one 4+3.

In the 32 FDRs providing previous measurements, five (16%) were ⩾4 ng ml^−1^ and eight (25%) were above the age-related cutoffs. No cancers were diagnosed in these men.

## DISCUSSION

The feasibility of studying the impact of screening on men who have a raised familial risk of prostate cancer will depend in part on the uptake rate and the workload involved in identifying eligible families, and in part on the sample size required to test hypotheses about the effects of screening. In this study, the uptake rates of ICs and FDRs, the workload associated with recruitment, and the referral rate and PPV of referral were studied.

The study demonstrated the importance of personal contact in clinic with prostate cancer patients, firstly to establish eligibility and secondly to discuss the study. In the clinic phase, 60% of ICs (685 out of 1140) reported no suitable relatives or relatives living abroad. Of the remaining 455, 85% agreed to receive the invitation letter. The higher uptake rates of ICs in this study in the clinic phase than in the postal phase is similar to that found in a US study contacting prostate cancer patients to collect data on family history of cancer (Plaetke *et al*, 2002). Reasons for non-participation of ICs included not having told their relatives that they had cancer, similar to our study.

In the clinic phase, the overall uptake rate of FDRs proceeding to a PSA test among those ICs with eligible relatives willing to receive the invitation letter was 41% (158 out of 389). The greatest loss occurred at the stage when ICs were asked to provide signed consent for their relative to be contacted (49%; 192 out of 389 consented). As ICs were encouraged to discuss the study with their relative, both men will have contributed to the non-response. Of those FDRs receiving an invitation letter, 87% (167 out of 192) consented to provide a PSA measurement, either present or previous, and 95% of the 167 proceeded to do so. First-degree male relatives were more likely to consent and proceed with screening if they were married/co-habiting than living with no partner (*P*<0.05): among FDRs who initially consented, 94% (190 out of 202) providing a PSA test were living with a partner compared with 78% (14 out of 18) who did not proceed. Data on socio-economic status were not collected. The higher detection of prostate cancer in non-manual than manual social class groups is partly considered to reflect difference in use of health service (Neal and Allgar, 2005), and similar trends are likely to be seen in relatives.

No Africans or African-Caribbeans participated in our study, although part of the London area would have included patients from this ethnic group. Some may have chosen not to take part and others may only have had relatives living abroad. These men remain an important group to study in the UK. In the UK, a higher prostate cancer mortality rate was found in West African and Caribbean migrants than in men born in England and Wales (Grulich *et al*, 1992). Research in the US shows that the risk of prostate cancer is higher in African Americans than in white Americans (Hsing *et al*, 2000), but the natural history is not understood fully (Freedland *et al*, 2000).

Other studies have shown that FDRs are less likely to be screened if they have high levels of anxiety (Bratt *et al*, 2000; Roumier *et al*, 2004) and had more than one affected FDR (Roumier *et al*, 2004). Psychosocial factors are explored further in the accompanying paper (refer Sweetman *et al*, 2006). Counselling of FDRs in our study was restricted because of the ethical requirement that they could not be contacted directly until the IC and FDR had given signed consent.

Limitations with our study include the fact that, in the clinic phase, the ascertainment rate of all potentially eligible ICs could not be estimated in all hospitals. There was a lack of routine standardised data sources in the hospitals to identify patients and their dates of appointments. As the NCRN staff often worked mainly in oncology, patients managed in urology could have been missed in some hospitals unless a urology or uro-oncology nurse specialist was involved. Also, patients on active surveillance who were being monitored by their GP and did not attend during the period of data collection would have been missed. The most complete ascertainment is likely to have occurred at RMH and Salisbury where a combination of clinic and postal methods was used to ensure that all potential eligible ICs on the hospital database were contacted.

The level of prostate cancer awareness and the rate of PSA testing already taking place in the UK will also have affected uptake in this study. Although ICs and FDRs were encouraged to participate even if the FDR had already been tested, it is uncertain what proportion of non-responders was eligible but had been previously screened. Previous testing was a reason for non-participation in a study of families with a high risk of prostate cancer in France (Roumier *et al*, 2004). In our study, 31% of FDRs reported having had a previous PSA test. In the accompanying psychosocial paper (refer Sweetman *et al*, 2006), it is reported that out of the 60% of FDRs who took part in the study, 41% (52 out of 128) had a blood test screening for prostate cancer. The true rate of prior screening cannot be accurately estimated from self-reported data, but the rate of prior testing in the study participants is certainly higher than that reported in the general UK population (Melia *et al*, 2004).

The study identified practical difficulties associated with identification of eligible patients and with arrangements for screening and referral. Without routine recording of family history of cancer, it is very difficult to identify eligible families. There was also a high workload associated with arranging the screening test and referral, as most GPs were only screening one patient and each GP practice needed support to follow the study protocol.

The percentage of PSA levels ⩾4 ng ml^−1^ in our study (10%) is similar to that reported in high-risk men in the Finnish arm of the European trial (Makinen *et al*, 2002) (11%). This percentage is higher than that in the general population: in the European general population screening trial in men with no family history (Makinen *et al*, 2002) screened in Finland and in men screened in Sweden (Hugosson *et al*, 2003), the percentages were 7.7 and 6.7% respectively in the prevalence round. In the study of high-risk families in France (Roumier *et al*, 2004), 5.7% of relatives had PSA levels >4 ng ml^−1^, but most of the relatives were sons below the age of 50 years. In the study of FDRs in families with ⩾2 brothers with prostate cancer in the USA (McWhorter *et al*, 1992), the proportion was 18%, but the FDRs belonged to families with a higher level of risk than in our study. In our study, the proportion was 16% (five out of 32) in FDRs providing a previous measurement, but the result may have been biased if the men who were worried by a previous test result were more likely to participate than those with lower readings.

The overall PPV of referral in our study (24%) was slightly lower than that reported in the Finnish study screening men with a family history of prostate cancer (Makinen *et al*, 2002) (27.6%) and was not significantly different from that in the general population (20%) (Roobol and Schroder, 2003). Although higher PPVs have been reported (33% (Matikainen *et al*, 1999) and 32% (Roumier *et al*, 2004)), these were in studies of relatives from families with ⩾2 relatives with prostate cancer, compared with ⩾1 in our study. The PPV in our study could have been affected by variation in the biopsy protocol between hospitals. Despite a standardised protocol, recommending eight biopsy cores to be taken, being sent to each FDR's consultant, in most cases the local protocol was used with a range of 6–12 cores. As the histological diagnosis of all men biopsied was checked and confirmed by the study review pathologist, observer variation in diagnosis and grading between pathologists is not expected to have affected the results.

Our study was not designed to have the power to study detection rates of prostate cancer. The level of risk from prostate cancer is expected to be lower than in families with ⩾2 FDRs with prostate cancer. Furthermore, families where the FDR had already been diagnosed with prostate cancer were excluded, and families of ICs who had died were not approached, so some families with perhaps a more aggressive form of the disease may have been excluded. It is not known what the uptake rate of screening would be in FDRs of ICs who had died from cancer.

Increased PSA testing in the general population has led to more men under the age of 60 years being tested both in the general population (Marotte *et al*, 2004; Hemminki *et al*, 2005) and in families with a history of the disease (Bock *et al*, 2005). Testing will bring forward the date of diagnosis and it will increase the diagnosis of slow-growing cancers, some of which may not be life threatening. Reports on the natural history of prostate cancer in high-risk men should consider the extent to which background PSA testing in the population and the selection criteria for families being studied may have influenced the results.

The effects of screening on prostate cancer mortality and quality of life in the general population and in high-risk groups remain uncertain. Most organisations, including the NHS Prostate Cancer Risk Management Programme, advise an informed choice approach if men request screening, so that they can weigh up the advantages and disadvantages (Hewitson and Austoker, 2005). The best evidence on the outcome of screening is likely to come from the randomised controlled trials. However, these were not designed and will not have the power to study adequately the effects of screening in high-risk groups. For the present, the impact of screening on these groups may have to be modelled using data from the general population.

In conclusion, these results have implications for the way that targeted prostate cancer PSA screening may be offered to high-risk groups. For example, there is a new study, IMPACT (The Identification of Men with genetic predisposition to ProstAte Cancer: Targeted screening in BRCA1/2 mutation carriers), and our study has informed the way that these men are invited into the study. While the results of the screening trials are awaited, PSA testing is likely to continue, and provision should be made for the counselling and provision of information to patients diagnosed at a young age to help them understand familial risk. Research is needed into the management of men with a very high risk of the disease, for example carriers of the BRCA2 gene (Edwards *et al*, 2003). Cancer networks could have a key role in ensuring that these men and their relatives are offered counselling. The study results emphasise the need for standardised data to be recorded nationally on the outcome of screening so that the effects of screening can be studied.

## Figures and Tables

**Table 1:** Summary of main sources for identification of index cases

**Data source**	**Date of diagnosis**	**Location**
Thames Cancer Registry	1998–2000	South Thames North Thames
South West Cancer Intelligence Service	1998–1999	South West Region
British Association of Urological Surgeons	1998–2001	Thames and South West Region
NCRN centres and urology clinics	RMH: January 1998–July 2004 Other: January 2000–July 2004	South West London, South West Region
Not in clinics, so posted invitation	RMH: January 1998–July 2004 Other: January 2000–July 2004	RMH and Salisbury

NCRN=National Cancer Research Network; RMH=Royal Marsden Hospital.

**Table 2:** The number of ICs and FDRs participating at different stages of the study

	**Postal phase**		**Clinic phase**		
**Stage**	**TCR and SWCIS**	**BAUS**	**Clinic contact only**	**No clinic contact, letter sent**	**Total**
Total number of ICs identified^a^	1368	319	1140	130	2957
Consultant reply and consent	515	60	Consultant consent prior to clinic contact	Consultant consent prior to clinic contact	
GP consent	340		N/A	117	
IC with eligible FDRs confirmed	N/A	12^b^	389	N/A	
IC consent returned with eligible FDR(s)	41	4	192	20	257
FDR consented to provide blood sample or previous test result, restricted to aged 45–69 years at the time of test	36	3	167	14	220
					
*PSA measurement*					
Study screen^c^	24	2	134	10	170
Previous screen	4	—	24	4	32

a Age ⩽65 years at diagnosis living in defined study areas, diagnosed 1998–2004.

b A total of 12 ICs only had their GP informed.

c Includes PSA measurements from 164 recorded at central laboratory and six recorded at the local laboratory of FDR.

Calculation of age of FDR in analysis: age of FDR at the date of PSA test. IC=index case; FDR=first-degree male relative; TCR=Thames Cancer Registry; SWCIS=South West Cancer Intelligence Service; BAUS: British Association of Urological Surgeons; GP=general practitioner; PSA=prostate-specific antigen.

**Table 3:** Results of PSA screening by the FDRs' reporting of history of previous PSA testing

					**PPV of referral^a^**	**PPV of biopsy^b^**
**Source of PSA test**	**No. of men screened**	**No. of men referred (%)**	**No. of men biopsied (%)**	**No. of men with cancer (%)**	**% (95% CI)**	**% (95% CI)**
Central+local lab, no prior PSA tests or not known	122	13 (10.7)	12 (9.8)	4 (3.3)	30.8 (9, 61)	33.3 (10, 65)
Central+local lab with prior PSA tests	48	8 (16.7)	4 (8.3)	1 (2.1)	12.5 (0, 53)	25.0 (1, 81)
Total	170	21 (12.4)	16 (9.4)	5 (2.9)	23.8 (8, 47)	31.2 (11, 59)

a The proportion of men diagnosed with prostate cancer on biopsy out of all men with a raised PSA who were recommended by the study protocol to be referred.

b The proportion of men diagnosed with prostate cancer on biopsy out of all men with a raised PSA who were recommended by the study protocol to be referred, and who actually proceeded to have a biopsy taken.

PSA=prostate-specific antigen; FDR=first-degree male relative; PPV=positive predictive value.
